# Analytical validation of hepatitis B core‐related antigen (HBcrAg) using dried blood spots (DBS)

**DOI:** 10.1111/jvh.13489

**Published:** 2021-03-01

**Authors:** Yusuke Shimakawa, Laura Vernoux, Audrey Gabassi, Séverine Mercier‐Delarue, Jeanne Perpétue Vincent, François Simon, Sarah Maylin

**Affiliations:** ^1^ Unité d'Épidémiologie des Maladies Émergentes Institut Pasteur Paris France; ^2^ Fujirebio Europe Gent Belgium; ^3^ Laboratoire de Virologie Hôpital Saint‐Louis AP‐HP Paris France

**Keywords:** analytical validation, diagnostic test, dried blood spot, hepatitis B core‐related antigen, resource‐limited country

## Abstract

Limited access to nucleic acid testing (NAT) to quantify HBV DNA levels, an essential tool to determine anti‐HBV treatment eligibility, represents a significant barrier to scale up HBV diagnostic services in resource‐limited countries. Hepatitis B core‐related antigen (HBcrAg) has the potential to become an affordable alternative because of its low cost (US$ <15/assay) and strong correlation with HBV DNA levels in treatment‐naïve patients. However, the current assay requires plasma or serum. To further facilitate its application to decentralized settings, we developed and evaluated a standardized procedure to quantify HBcrAg using dried blood spots as a tool to diagnose HBV‐infected people with high viraemia. We evaluated the following elution method optimized to quantify HBcrAg: suspension of a punched blood‐soaked disc (11 mm) of Whatman 903 Protein Saver Card in 450 µL of PBS 0.05% Tween 20, followed by an incubation for 4 h at room temperature and a centrifugation at 10,000 *g* for 10 minutes. 150 µL of DBS eluate was used to quantify HBcrAg using chemiluminescent enzyme immunoassay (LUMIPULSE^®^ G600II, Fujirebio). The limit of detection of dried blood spot HBcrAg in relation with HBV DNA levels was 19,115 IU/mL across the five major HBV genotypes (A/B/C/D/E). A strong linear correlation was confirmed between dried blood spot HBcrAg and HBV DNA levels (*r* = 0.94, *p* < 0.0001) in samples with high viral loads (range: 3.7–7.0 log IU/mL). The coefficient of variation ranged between 4.0–11.2% for repeatability and 3.9–12.2% for reproducibility. Analytical specificity was 100% (95% CI: 83.9–100%) in HBV‐negative samples. Using our elution method, it may be possible to identify HBV‐infected patients with high viraemia who need antiviral therapy using dried blood spot and HBcrAg. A large‐scale clinical validation is warranted in resource‐limited countries.

## 1 INTRODUCTION

Hepatitis B virus (HBV) infection is a major global health concern, and has been targeted by the United Nation's Sustainable Development Goals. In 2016, the World Health Organization (WHO) developed a strategy to globally eliminate viral hepatitis as a public health threat by 2030, and one of the key service targets has been to increase the uptake of antiviral therapy in people with chronic HBV infection (CHB) from 8% (2015) to 80% (2030).^1^ Anti‐HBV treatment is only indicated in those who have a combination of high HBV DNA levels and active liver inflammation, those with both high HBV DNA levels and significant liver fibrosis, or those who already developed cirrhosis.^2^, ^3^, ^4^, ^5^ It is therefore crucial to ensure the widest possible access to diagnostic tools that are essential to evaluate the eligibility for antiviral therapy in order to reach the elimination goal.

However, >95% of HBV‐infected people live in low‐income and middle‐income countries (LMICs),^6^ and they have a seriously limited access to these diagnostic tests, especially nucleic acid test (NAT) to quantify HBV DNA levels. Real‐time polymerase chain reaction (RT‐PCR), a standard NAT for HBV DNA quantification, is expensive (US$ 20‐130/assay),^7^ often restricted to sophisticated urban laboratories, and requires highly skilled laboratory technicians to avoid the risk of contamination and to make a correct interpretation of the results.^8^, ^9^ Consequently, WHO explicitly acknowledges a pressing need for a low‐cost, simple assay to identify HBV‐infected people with high viral load.^10^

Hepatitis B core‐related antigen (HBcrAg) is a novel serological marker to measure hepatitis B viral replication, manufactured by Fujirebio (Tokyo, Japan).^11^ The assay quantifies hepatitis B core antigen (HBcAg), e antigen (HBeAg), as well as p22cr and c‐terminal modified HBcAg contained in the empty particle fraction in blood, irrespective of anti‐HBc or anti‐HBe antibodies.^12^ A recent systematic review and meta‐analysis found that in treatment‐naïve patients with CHB, serum HBcrAg levels are closely correlated with serum HBV DNA levels irrespective of viral genotypes,^13^ suggesting that HBcrAg might be a credible alternative to RT‐PCR to identify HBV‐infected people in need of antiviral therapy.^14^ Moreover, the reagent for HBcrAg assay is inexpensive (US$ <15/assay) and its assay is simpler to perform compared with the conventional RT‐PCR, which makes this sero‐marker suitable for LMICs to decentralize clinical staging and anti‐HBV treatment services.

However, the current operational characteristics of HBcrAg assay do not allow its immediate integration into the primary care system in LMICs. First, its quantification requires automated chemiluminescent enzyme immunoassay (CLEIA) platform (LUMIPULSE^®^). Although the machine costs less (US$ <20,000) than other conventional CLEIA instruments, its installation in a peripheral laboratory is unrealistic given the need for regular maintenance and a reliable electricity supply. Second, the assay requires serum or plasma as a specimen type. Venepuncture at a primary care setting and shipment of frozen serum/plasma to a central laboratory is unlikely to be feasible because primary care facilities often lack a centrifuge and freezer. Alternatively, the use of dried blood spot (DBS) may largely overcome these problems. Even in a primary care clinic, finger‐prick capillary blood can be collected easily and less invasively on a filter paper, which can then be shipped to a central laboratory at a room temperature. This avoids referral of HBV‐infected patients to a central laboratory, and enables their clinical staging at the primary care level.^15^, ^16^ We therefore developed a standardized elution procedure to quantify HBcrAg using DBS specimen, and conducted an analytical validation of the use of DBS for HBcrAg assay as a tool to diagnose high HBV DNA levels which warrant an initiation of antiviral therapy.

## 2 MATERIALS AND METHODS

### 2.1 Preparation for DBS specimens

Blood samples from HBV‐infected patients collected in EDTA K2 tube (BD Vacutainer^®^) are routinely sent to the laboratory of virology at Saint Louis Hospital for HBV DNA measurement. A total of five leftover samples were used for this study; from each of the five major viral genotypes (A/B/C/D/E), we selected one plasma sample with high HBV DNA levels (>7.0 log IU/mL), high HBcrAg levels (>7.0 log U/mL) and negative for antibody to hepatitis C virus (HCV) and anti‐HIV antibody. Plasma HBV DNA levels were quantified using a commercial RT‐PCR (Cobas^®^ 6800, Roche Diagnostics, Mannheim, Germany). Plasma HBcrAg levels were quantified using CLEIA (LUMIPULSE^®^ G600II, Fujirebio Europe, Gent, Belgium). Anti‐HCV and anti‐HIV were tested using ARCHITECT i4000 system (Abbott Laboratories, Rungis, France). These plasma samples were spiked into whole blood specimens from a donor non‐infected with HBV (negative for hepatitis B surface antigen (HBsAg) and negative for HBV DNA) to obtain serially diluted concentrations of HBV DNA at 10^7^, 10^6^, 10^5^, 10^4^, 5000, 2000, 1000, 500 and 200 IU/mL. Then, these whole blood specimens spiked with an infected plasma were used to prepare DBS, which was specifically made for each of the five viral genotypes and each of the nine different HBV DNA concentrations. One spot consisted of 50 μL of spiked whole blood, dropped onto a circle of a filter paper recommended by the WHO (Whatman 903, Protein Saver Card, Whatman/GE Healthcare, Springfiled Mill, UK).^17^ The cards were left to dry for at least three hours in a horizontal position. We also prepared blank DBS specimens using non‐infected whole blood without spiking with the infected plasma. All DBS were stored at room temperature. This work was exempt from ethical approval because the analysis only used anonymized remnant leftover samples from previous clinical studies.

### 2.2 Optimization for preparing DBS eluate

To optimize the method to prepare DBS eluate, we examined the following operation parameters: (i) elution buffer (phosphate buffered saline (PBS) with 0.05% Tween 20 versus pre‐treatment solution for HBcrAg (Fujirebio); (ii) number of dried blood spots (1 versus 2 circles); (iii) volume of elution buffer (from 300 µL to 600 µL); and (iv) incubation condition (4°C overnight versus room temperature for 4 hours). Using the DBS samples with the HBV DNA concentration of 10^4^ IU/mL, HBcrAg levels were compared according to these operational parameters. We found that the elution from one spot (compared to two spots) and PBS 0.05% Tween 20 (compared to pre‐treatment solution) resulted in higher rate of HBcrAg detection, whilst no difference was observed according to the amount of elution buffer or incubation condition (data not shown). Consequently, we defined the following optimal procedure:

Step 1: Punch out one disc (11 mm, containing 50 µL of blood) from a blood‐soaked circle of the 903 filter card. Before punching the next filter paper, the puncher was cleaned with the surface decontaminant DNA AWAY™ (Thermo Fisher Scientific, Waltham, MA, USA).
Step 2: Suspend the disc in a 24‐wells microplate with 450 µL of PBS 0.05% Tween 20.
Step 3: Incubate 4 hours at room temperature with continuous agitation.
Step 4: Remove the disc, transfer to a tube and centrifuge the tube at 10,000 *g* for 10 minutes.
Step 5: Process 150 µL of DBS eluate for the pre‐treatment and quantify HBcrAg using LUMIPULSE^®^ CLEIA (Fujirebio).

### 2.3 Quantification of HBcrAg

One hundred fifty microliter of DBS eluate was analysed for HBcrAg using a fully automated CLEIA (LUMIPULSE^®^ G600II) according to the manufacturer's instructions. Briefly, samples were pre‐treated with sodium dodecyl sulphate, followed by incubation with monoclonal antibodies against denatured HBcAg and HBeAg. HBcrAg concentrations were calculated from a standard curve generated using recombinant HBeAg and are expressed in kU/mL. According to the manufacturer, the analytical sensitivity for HBcrAg using plasma or serum is 1 kU/mL (3.0 log U/mL), and the measurement range is from 1 to 10,000 kU/mL (3.0–7.0 log U/mL). Samples above 7.0 log U/mL were retested after the dilution at 1/100 with a sample diluent provided by the manufacturer.

### 2.4 Limit of detection of DBS HBcrAg

In order to ensure that an analytical signal generated by a blank specimen not containing HBcrAg does not overlap with a signal generated by a low‐concentration specimen, we used 11 HBV‐negative spots, and 20 spots with a low HBcrAg concentration (3.0 log U/mL in blood) infected with HBV genotype A. For each type of specimen, we obtained means and standard deviations (SD) of HBcrAg levels using DBS. Then, the limit of blank (LOB) and limit of detection (LOD) were estimated by the following equations^18^:

$$\text{Limit of blank (LOB)}={\text{mean}}_{\text{blank}}+1.645\times {\text{SD}}_{\text{blank}}$$

$$\text{Limit of detection (LOD)}=\text{LOB}+1.645\times {\text{SD}}_{\text{low - concentration sample}}$$

### 2.5 Analytical validation of DBS HBcrAg using HBV DNA RT‐PCR as reference

In order to assess the use of DBS HBcrAg as a tool to identify patients with high HBV DNA levels, we compared the index test (HBcrAg using DBS) against a reference test (HBV DNA quantified by RT‐PCR) for the following outcomes: linear range, limit of detection, repeatability, reproducibility and analytical specificity.

At each of the nine different HBV DNA concentrations, HBcrAg measurement using DBS was repeated at least in triplicate in a single run. In addition, for the viral loads of 10^7^, 10^5^, 5000, 2000 and 1000 IU/mL, the measurement was repeated in 20 replicates in a single run. For each of the five major genotypes (A‐E), the levels of HBcrAg quantified using DBS specimens were plotted against the corresponding concentrations of HBV DNA in blood; then the correlation coefficient was deducted. Using a probit analysis, the LOD in relation to HBV DNA level in blood was defined as a concentration that can be detectable in 95% of time (C_95_).

Repeatability and reproducibility were assessed at three HBV DNA levels (10^7^, 10^5^ and 2000 IU/mL) for each of the five genotypes. The repeatability was assessed by repeating the measurement for a total of 20 times at each concentration in a single run in a day. The reproducibility was evaluated by repeating the measurement in duplicate in one run per day over 20 days. Finally, cross‐reactivity was evaluated in samples negative for HBV (HBsAg‐negative and DNA negative), but positive for HCV (anti‐HCV positive, n = 10) or HIV (anti‐HIV positive, n = 11). Statistical analyses were performed using STATA 13.0 (Stata Corporation, College Station, Texas).

## 3 RESULTS

### 3.1 Analytical sensitivity of DBS HBcrAg

In 11 spots from a blank DBS specimen, HBcrAg was detected at the levels lower than the limit of detection as per the manufacturer's instruction for plasma or serum (<3.0 log U/mL) and varied between 2.0 and 2.3 log U/mL (mean 2.19 log U/mL ±0.15 log U/mL), providing the limit of blank of 2.44 log U/mL. In 20 low concentration DBS specimens, SD of mean HBcrAg using DBS was 0.19. This gave a LOD of 2.75 log U/mL. To make consistent with the manufacturer's instruction for plasma/serum samples, we defined the analytical sensitivity of DBS HBcrAg to be 3.0 log U/mL.

### 3.2 Limit of detection of DBS HBcrAg in relation to HBV DNA levels

Using the probit analysis, the LOD (C_95_) of DBS HBcrAg in relation to HBV DNA levels for all genotypes (A‐E) was estimated at 4.3 log IU/mL (19,115 IU/mL) (Table 1). The LOD for each genotype was as follows: 4.6 log IU/mL (genotype A); 3.6 log IU/mL (genotype B); 4.2 log IU/mL (genotype C); 4.3 log IU/mL (genotype D) and 4.6 log IU/mL (genotype E).

**TABLE 1:** Probit analysis for HBcrAg detection using DBS by the level of HBV DNA (all genotypes: A‐E, n = 310)

HBV DNA levels		No. of replicates tested	No. of replicates detected by DBS HBcrAg	% detected
IU/mL	Log IU/mL			
10,000,000	7.0	80	80	100
1,000,000	6.0	15	15	100
100,000	5.0	100	100	100
10,000	4.0	15	6	40
1000	3.0	100	0	0

Abbreviations: DBS HBcrAg, hepatitis B core‐related antigen measured using dried blood spots; HBV, hepatitis B virus.

### 3.3 Linear range

**FIGURE 1:**
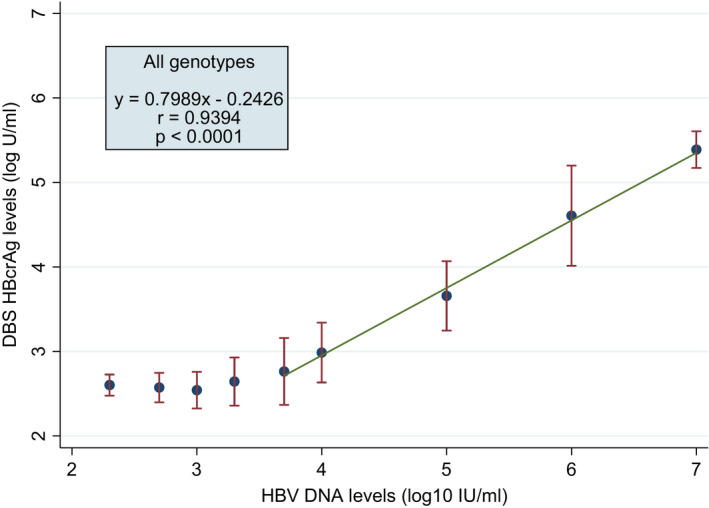
Linear relationship between DBS HBcrAg levels and HBV DNA levels

Figure 1 shows the linear range of DBS HBcrAg in relation with HBV DNA levels. The linearity was observed between the HBV DNA level of 3.7 log IU/mL (ie 5,000 IU/mL) and 7.0 log IU/mL. In this range of HBV DNA concentrations, the linear regression analysis gave the following equation:

y=0.7989x‐0.2426

There was a strong correlation between DBS HBcrAg levels and viral loads (Pearson's correlation coefficient: *r* = 0.94, *p* < 0.0001).

### 3.4 Repeatability & reproducibility

Table 2 shows that the coefficient of variation ranged between 4.0–11.2% for repeatability and 3.9–12.2% for reproducibility across the different genotypes. Table 3 presents the coefficients of variation by viral genotype; we did not identify any difference between the genotypes.

**TABLE 2:** Repeatability (n = 280) and reproducibility (n = 561)

Blood HBV DNA level (IU/mL)	Repeatability					Reproducibility				
	N	Mean HBcrAg (log U/mL)	SD	% CV	95% CI	N	Mean HBcrAg (log U/mL)	SD	% CV	95% CI
2000	100	2.64	0.28	10.8	2.59–2.70	201	2.68	0.32	11.8	2.63–2.72
10^5^	100	3.66	0.41	11.2	3.58–3.74	200	3.69	0.45	12.2	3.63–3.75
10^7^	80	5.39	0.22	4.0	5.34–5.44	160	5.42	0.21	3.9	5.39–5.45

Abbreviations: 95% CI, 95% confidence interval; CV, coefficient of variation; HBcrAg, hepatitis B core‐related antigen; HBV, hepatitis B virus; SD, standard deviation.

**TABLE 3:** Repeatability and reproducibility by viral genotypes

Blood HBV DNA level (IU/mL)	Repeatability					Reproducibility				
	N	Mean HBcrAg (log U/mL)	SD	% CV	95% CI	N	Mean HBcrAg (log U/mL)	SD	% CV	95% CI
Genotype A										
2000	20	2.52	0.19	7.4	2.43–2.60	41	2.54	0.25	9.8	2.46–2.62
10^5^	20	3.36	0.07	2.0	3.32–3.39	40	3.33	0.09	2.6	3.30–3.35
10^7^	20	5.25	0.05	1.0	5.22–5.27	40	5.28	0.12	2.2	5.24–5.32
Genotype B										
2000	20	2.92	0.18	6.2	2.83–3.00	40	2.99	0.09	2.9	2.99–3.16
10^5^	20	4.44	0.05	1.1	4.42–4.46	40	4.53	0.12	2.7	4.49–4.56
10^7^	0	N/A	N/A	N/A	N/A	N/A	N/A	N/A	N/A	N/A
Genotype C										
2000	20	2.60	0.09	3.6	2.55–2.64	40	2.64	0.33	12.4	2.54–2.75
10^5^	20	3.53	0.18	5.1	3.44–3.61	40	3.68	0.09	2.5	3.65–3.71
10^7^	20	5.25	0.05	1.0	5.22–5.27	40	5.67	0.11	2.0	5.63–5.70
Genotype D										
2000	20	2.32	0.14	6.0	2.25–2.39	40	2.66	0.37	13.9	2.54–2.78
10^5^	20	3.56	0.05	1.4	3.53–3.58	40	3.56	0.11	3.2	3.52–3.59
10^7^	20	5.46	0.08	1.5	5.42–5.50	40	5.49	0.14	2.4	5.44–5.53
Genotype E										
2000	20	2.88	0.25	8.6	2.76–2.99	40	2.55	0.25	9.7	2.47–2.62
10^5^	20	3.41	0.04	1.3	3.39–3.43	40	3.35	0.11	3.2	3.32–3.39
10^7^	20	5.16	0.08	1.5	5.12–5.20	40	5.24	0.14	2.8	5.19–5.29

Abbreviations: 95% CI, 95% confidence interval; CV, coefficient of variation; HBcrAg, hepatitis B core‐related antigen; HBV, hepatitis B virus; SD, standard deviation.

### 3.5 Analytical specificity

Using DBS, HBcrAg was not detected in any of samples negative for HBV and positive for anti‐HCV antibody (n = 10), and any of samples negative for HBV and positive for anti‐HIV antibody (n = 11). This provided a specificity of 100% (95% CI: 83.9–100%).

## 4 DISCUSSION

We optimized a procedure to prepare DBS eluate for HBcrAg quantification. Using our methods, we found that the LOD of DBS HBcrAg in relation with HBV DNA levels was 19,115 IU/mL across the five major HBV genotypes. We also found a strong correlation between DBS HBcrAg levels and HBV DNA levels (*r* = 0.94, *p* < 0.0001) in those with high viral loads (range: 3.7–7.0 log IU/mL), as well as satisfactory results on linear range, repeatability, reproducibility and specificity.

Although this procedure cannot be used for a detection of small quantities of HBV DNA, HBcrAg using DBS can identify clinically important high viral loads. It is particularly relevant because the LOD in relation to HBV DNA levels corresponded well with a viral load threshold of 20,000 IU/mL, a cut‐off value to consider initiating antiviral therapy in people with chronic HBV infection according to the WHO guidelines.^2^ Another likely application of DBS HBcrAg is categorizing pregnant women found to carry HBsAg during antenatal care screening for HBV. HBV‐infected women with a viral load of ≥200,000 IU/mL should receive antiviral prophylaxis during the third trimester of pregnancy in order to prevent HBV MTCT.^19^, ^20^, ^21^ Considering the poor availability of RT‐PCR in LMICs where out‐of‐pocket payment for health care is frequent, the use of DBS HBcrAg may render such a strategy to prevent MTCT more realistic.

To our knowledge this is the first study to evaluate the use of DBS for HBcrAg quantification. DBS has been used in the medical field since the 1960s.^22^ Previously, this was primarily used to facilitate newborn screening, but nowadays this is largely exploited in the field of infectious diseases in LMICs due to the convenience that this offers for collection, transport and storage of blood samples. Whole blood can be collected with a minimal invasion (a finger‐prick capillary blood) and do not require a cold chain. DBS has been widely used with molecular diagnostics, and it has been shown to increase the uptake of screening for hepatitis B, C and for HIV among special populations.^23^, ^24^, ^25^

For the diagnosis of HCV viraemia, the WHO has made a conditional recommendation to use hepatitis C core antigen, a serological marker well correlated with HCV RNA levels, in areas where there is limited access to NAT.^10^ To further facilitate the decentralization of HCV testing services, a study in Tanzania evaluated the performance of the use of DBS to quantify HCV core antigen levels, and found a good correlation between the levels of HCV core antigen using DBS and serum HCV RNA viral loads.^26^ For the diagnosis of HBV, the detection of HBsAg from DBS was found highly performant in a systematic review.^15^ Similarly, the use of DBS to detect and quantify HBV DNA levels has been validated although the number and quality of studies are still limited.^16^ Our results indicate that HBcrAg can be also accurately detected and quantified using the DBS sample.

In treatment‐naïve HBV‐infected patients, a positive association has been observed between the levels of serum HBcrAg and the degree of liver diseases (elevated alanine aminotransferase, significant fibrosis), suggesting that serum HBcrAg might be useful to identify HBV‐infected patients in need of antiviral therapy.^13^, ^14^ Moreover, several longitudinal studies from Asia suggested that HBcrAg might be even more accurate than HBV DNA levels to predict liver disease progression, including cirrhosis and HCC, in treatment‐naïve patients.^27^, ^28^, ^29^ Nevertheless, its utility has been recognized far more prominently among HBV‐infected patients treated with nucleos(t)ide analogues who achieved undetectable serum HBV DNA.^30^ HBcrAg has been proposed as a marker of intrahepatic covalently closed circular DNA (cccDNA), the viral DNA pool responsible for the chronicity of HBV infection, which cannot be reached by the nucleos(t)ide analogues.^31^ Therefore, HBcrAg could be of help in monitoring the genuine viral replication of HBV‐infected patients under the nucleos(t)ide analogues, and in predicting the prognosis of these patients. Although the current analytical sensitivity of the DBS HBcrAg may not be optimal for this objective, the limit of detection and quantification of serum HBcrAg has been recently improved from 3.0 log U/mL to 2.1 log U/mL using a highly sensitive assay developed by the same manufacturer (iTACT‐HBcrAg, Fujirebio, Japan).^32^, ^33^ The application of this newer assay to the DBS specimens might overcome the limited analytical sensitivity of the DBS HBcrAg.

Recently, Cepheid (US) commercialized a point‐of‐care HBV DNA PCR kit (Xpert^®^), and a few studies validated its diagnostic performance in resource‐limited countries.^34^ According to the Foundation for Innovative New Diagnostics (FIND), the negotiated price of Xpert HBV viral load cartridge in LMICs is US$ 14.90 per assay, and that of a standard four‐module system is US$ 17,500.^35^ Although the Xpert platform has been made widely available in many LMICs thanks to the tuberculosis programmes, it remains unknown whether patients with HBV infection can have unrestricted access to these assays. Although HBcrAg can be quantified under a competitive price (US$ <15/assay and CLEIA costs US$ <20,000), its CLEIA platform (LUMIPULSE^®^) is still unavailable in many LMICs including sub‐Saharan Africa; its product roll‐out in resource‐limited settings is therefore urgently needed. The adoption of DBS sampling can allow the specimens collected at a primary care setting to be batch tested at a centralized laboratory, eliminating the need for a LUMIPULSE^®^ system in each peripheral laboratory. Alternatively, an out‐licensing of HBcrAg to other CLEIA platforms already available in these countries, or the development of an inexpensive rapid diagnostic test based on lateral‐flow technology, could also overcome the limited access to the LUMIPULSE^®^ platform in LMICs.

The study has limitations. First, DBS were artificially prepared using non‐infected whole blood spiked with HBV‐infected plasma samples. Ideally, DBS should have been directly prepared using a finger‐prick capillary whole blood from infected people. Second, we only assessed analytical sensitivity of the use of DBS. We therefore plan to evaluate diagnostic sensitivity and specificity of HBcrAg using DBS with a finger‐prick capillary blood in a real‐life field condition in LMICs. Third, we did not assess whether the performance of HBcrAg using DBS varies by the presence of HBeAg given the insufficient volume of the remnant samples. This should be evaluated in a future study.

In conclusion, the quantification of HBcrAg from DBS eluate might be a promising tool to detect clinically important HBV viraemia. This may particularly benefit HBV elimination efforts in LMICs by allowing wider coverage of chronic HBV infection staging even for those living in remote rural areas.

## CONFLICT OF INTEREST

LV is an employee of Fujirebio Europe. All other authors have no conflict of interest.

## References

[1] WHO . Global Health Sector Strategy on Viral Hepatitis 2016–2021. Geneva, Switzerland; 2016.

[2] WHO . Guidelines for the prevention, care and treatment of persons with chronic hepatitis B infection. 2015;(March):166. 10.1186/1471-2334-13-288 26225396

[3] Lampertico P , Agarwal K , Berg T , et al. EASL 2017 Clinical Practice Guidelines on the management of hepatitis B virus infection. J Hepatol. 2017;67(2):370‐398. 10.1016/j.jhep.2017.03.021 28427875

[4] Terrault NA , Bzowej NH , Chang K‐M , Hwang JP , Jonas MM , Murad MH . AASLD guidelines for treatment of chronic hepatitis B. Hepatology. 2016;63(1):261‐283. 10.1002/hep.28156. 26566064 PMC5987259

[5] Sarin SK , Kumar M , Lau GK , et al. Asian‐Pacific clinical practice guidelines on the management of hepatitis B: a 2015 update. Hepatol Int. 2016;10(1):1‐98. 10.1007/s12072-015-9675-4. PMC4722087 26563120

[6] Razavi‐Shearer D , Gamkrelidze I , Nguyen MH , et al. Global prevalence, treatment, and prevention of hepatitis B virus infection in 2016: a modelling study. Lancet Gastroenterol Hepatol. 2018;3(6):383‐403. 10.1016/S2468-1253(18)30056-6. 29599078

[7] Tordrup D , Hutin Y , Stenberg K , et al. Additional resource needs for viral hepatitis elimination through universal health coverage: projections in 67 low‐income and middle‐income countries, 2016–30. Lancet Glob Heal. 2019;7(9):e1180‐e1188. 10.1016/S2214-109X(19)30272-4. 31353061

[8] Andriamandimby SF , Olive MM , Shimakawa Y , et al. Prevalence of chronic hepatitis B virus infection and infrastructure for its diagnosis in Madagascar: Implication for the WHO’s elimination strategy. BMC Public Health. 2017;17(1):636. 10.1186/s12889-017-4630-z 28778194 PMC5544978

[9] Ishizaki A , Bouscaillou J , Luhmann N , et al. Survey of programmatic experiences and challenges in delivery of hepatitis B and C testing in low‐ and middle‐income countries. BMC Infect Dis. 2017;17(Suppl 1):693. 10.1186/s12879-017-2767-0 29143609 PMC5688462

[10] World Health Organization . Guidelines on Hepatitis B and C Testing. 2017. 10.1007/s00216-014-7926-1.

[11] Kimura T , Rokuhara A , Sakamoto Y , et al. Sensitive enzyme immunoassay for hepatitis B virus core‐related antigens and their correlation to virus load. J Clin Microbiol. 2002;40(2):439‐445. 10.1128/JCM.40.2.439-445.2002. 11825954 PMC153363

[12] Ning X , Basagoudanavar SH , Liu K , et al. Capsid phosphorylation state and hepadnavirus virion secretion. J Virol. 2017;91(9):e00092‐17. 10.1128/JVI.00092-17 28228589 PMC5391479

[13] Yoshida K , Desbiolles A , Feldman SF , et al. Hepatitis B core‐related antigen to indicate high viral load: systematic review and meta‐analysis of 10,397 individual participants. Clin Gastroenterol Hepatol. 2021;19(1):46‐60.e8. 10.1016/j.cgh.2020.04.045. 32360825

[14] Shimakawa Y , Ndow G , Njie R , et al. Hepatitis B core‐related antigen: an alternative to hepatitis B virus DNA to assess treatment eligibility in Africa. Clin Infect Dis. 2020;70(7):1442‐1452. 10.1093/cid/ciz412. 31102406

[15] Lange B , Cohn J , Roberts T , et al. Diagnostic accuracy of serological diagnosis of hepatitis C and B using dried blood spot samples (DBS): two systematic reviews and meta‐analyses. BMC Infect Dis. 2017;17(Suppl 1):700. 10.1186/s12879-017-2777-y 29143672 PMC5688450

[16] Lange B , Roberts T , Cohn J , et al. Diagnostic accuracy of detection and quantification of HBV‐DNA and HCV‐RNA using dried blood spot (DBS) samples ‐ a systematic review and meta‐analysis. BMC Infect Dis. 2017;17(Suppl 1):693. 10.1186/s12879-017-2776-z 29143616 PMC5688458

[17] WHO . WHO manual for HIV drug resistance testing using dried blood spot specimens. 2012.

[18] Burd EM . Validation of laboratory‐developed molecular assays for infectious diseases. Clin Microbiol Rev. 2010;23(3):550‐576. 10.1128/CMR.00074-09. 20610823 PMC2901657

[19] Funk AL , Lu Y , Yoshida K , et al. Efficacy and safety of antiviral prophylaxis during pregnancy to prevent mother‐to‐child transmission of hepatitis B virus: a systematic review and meta‐analysis. Lancet Infect Dis. 2021;21(1):70‐84. 10.1016/S1473-3099(20)30586-7. 32805200

[20] Boucheron P , Lu Y , Yoshida K , et al. Accuracy of HBeAg to identify pregnant women at risk of transmitting hepatitis B virus to their neonates: a systematic review and meta‐analysis. Lancet Infect Dis. 2021;21(1):85‐96. 10.1016/S1473-3099(20)30593-4. 32805201

[21] WHO . Prevention of mother‐to‐child transmission of hepatitis B virus: guidelines on antiviral prophylaxis in pregnancy. 2020. http://www.ncbi.nlm.nih.gov/pubmed/32833415 32833415

[22] Guthrie R , Susi A . A simple phenylalanine method for detecting phenylketonuria in large populations of newborn infants. Pediatrics. 1963;32:338‐343. 14063511

[23] Keel P , Edwards G , Flood J , et al. Assessing the impact of a nurse‐delivered home dried blood spot service on uptake of testing for household contacts of hepatitis B‐infected pregnant women across two London trusts. Epidemiol Infect. 2016;144(10):2087‐2097. 10.1017/S0950268815003325. 26833270 PMC9150565

[24] Coats JT , Dillon JF . The effect of introducing point‐of‐care or dried blood spot analysis on the uptake of hepatitis C virus testing in high‐risk populations: A systematic review of the literature. Int J Drug Policy. 2015;26(11):1050‐1055. 10.1016/j.drugpo.2015.05.001. 26118799

[25] Sirirungsi W , Khamduang W , Collins IJ , et al. Early infant HIV diagnosis and entry to HIV care cascade in Thailand: an observational study. Lancet HIV. 2016;3(6):e259‐e265. 10.1016/S2352-3018(16)00045-X. 27240788 PMC6047735

[26] Mohamed Z , Mbwambo J , Shimakawa Y , et al. Clinical utility of HCV core antigen detection and quantification using serum samples and dried blood spots in people who inject drugs in Dar‐es‐Salaam, Tanzania. J Int AIDS Soc. 2017;20(1):21856. 10.7448/IAS.20.1.21856. 28953324 PMC5964737

[27] Tada T , Kumada T , Toyoda H , et al. HBcrAg predicts hepatocellular carcinoma development: An analysis using time‐dependent receiver operating characteristics. J Hepatol. 2016;65(1):48‐56. 10.1016/j.jhep.2016.03.013. 27034253

[28] Tada T , Kumada T , Toyoda H , Kobayashi N , Akita T , Tanaka J . Hepatitis B virus core‐related antigen levels predict progression to liver cirrhosis in hepatitis B carriers. J Gastroenterol Hepatol. 2018;33(4):918‐925. 10.1111/jgh.13989. 28914957

[29] Tseng T‐C , Liu C‐J , Hsu C‐Y , et al. High level of hepatitis B core‐related antigen associated with increased risk of hepatocellular carcinoma in patients with chronic HBV infection of intermediate viral load. Gastroenterology. 2019;157(6):1518‐1529.e3. 31470004 10.1053/j.gastro.2019.08.028

[30] Wong DK‐H , Seto W‐K , Cheung K‐S , et al. Hepatitis B virus core‐related antigen as a surrogate marker for covalently closed circular DNA. Liver Int. 2017;37(7):995‐1001. 10.1111/liv.13346. 27992681

[31] Mak LY , Wong DKH , Cheung KS , Seto WK , Lai CL , Yuen MF . Review article: hepatitis B core‐related antigen (HBcrAg): an emerging marker for chronic hepatitis B virus infection. Aliment Pharmacol Ther. 2018;47(1):43‐54. 10.1111/apt.14376. 29035003

[32] Inoue T , Matsuura K , Iio E , et al. Clinical effectiveness of a novel fully automated high‐sensitive hepatitis B core‐related antigen assay for monitoring nucleos(t)ide analogues therapy in hepatitis B envelope antigen‐negative patients. Hepatology. 2020;72(suppl):725.

[33] Inoue T , Kusumoto S , Ogawa S , et al. Clinical effectiveness of a novel high‐sensitive hepatitis B core‐related antigen assay for early detection of hepatitis B virus reactivation. Hepatology. 2020;72(suppl):726.

[34] Woldemedihn GM , Rueegg CS , Desalegn H , Aberra H , Berhe N , Johannessen A . Validity of a point‐of‐care viral load test for hepatitis B in a low‐income setting. J Virol Methods. 2021;289:114057. 10.1016/j.jviromet.2020.114057. 33359613

[35] Foundation for Innovative New Diagnostics . Negotiated Prices. https://www.finddx.org/pricing/genexpert/. Accessed February 1, 2021

